# Hydraulic Conductivity of Saturated Soil Medium through Time-Domain Reflectometry

**DOI:** 10.3390/s20237001

**Published:** 2020-12-07

**Authors:** Seungjae Lee, Hyung-Koo Yoon

**Affiliations:** Department of Construction and Disaster Prevention Engineering, Daejeon University, Daejeon 34520, Korea; seungjae1215@edu.dju.ac.kr

**Keywords:** dielectric constant, hydraulic conductivity, laboratory test, time-domain reflectometry

## Abstract

Time-domain reflectometry (TDR) has been extensively used to study soil behaviors. The objective of this study is to propose a method for measuring hydraulic conductivity using TDR. The dielectric constant deduced from TDR is influenced by the electrical resistance of the medium, and it can be converted into the electrical resistivity of the material. Thus, the theoretical relationship between the dielectric constant and hydraulic conductivity is established because electrical resistivity is a function of hydraulic conductivity. A cell is developed for measuring both the dielectric constant and hydraulic conductivity simultaneously. Three electrodes are used to measure the reflected waveform by using the principle of TDR. The following specimens are used to verify the proposed technique: glass beads, Jumunjin sand, and soil extracted from a field. The dielectric constant is converted into hydraulic conductivity, and it is compared with the value determined by a constant-head experiment for reference. The comparison shows a high similarity. Verification is also carried out through field experiments. This study demonstrates that the proposed method is an alternative method to find the hydraulic conductivity through TDR.

## 1. Introduction

Water can permeate soil through pores. Water-flow rate is an essential parameter used to understand the soil behavior for land management. Hydraulic conductivity, one of the engineering parameters used to characterize water flow, is defined as the ratio of the flow rate (flux) to the potential gradient in the unit of [LT^−1^]. Various experimental methods have been used for the measurement of hydraulic conductivity, including pumping, double-tube, and infiltrometry in-situ methods and constant head, falling head, and empirical laboratory methods. Generally, field tests provide highly reliable values. However, these tests are uneconomical due to high costs and time consumption. Therefore, an additional technique is required to measure hydraulic conductivity with increased reliability based on efficient experimental procedure and analysis.

Nondestructive methods have been extensively used to measure the characteristics of an object because they are simple and economical. Time-domain reflectometry (TDR) is one of the nondestructive techniques that can be used to measure the dielectric constant of a material through the reflected electromagnetic wave; it is one of the most popular techniques for understanding the properties of materials. The theoretical concept of TDR was introduced by Fellner–Feldegg [1]. The dielectric constant of a material, commonly referred to as relative permittivity, is defined as the ratio of the permittivity of the material to the permittivity of vacuum; it is an inherent property of a material attributed to the electrical charges in an electromagnetic field. The dielectric constant of water is higher (ε = 80 at 20 °C) than that of soil constituents such as rock, gravel, sand, and silt (ε ≈ 2.5–15); therefore, it is possible to detect the amount of water in soil [2] through TDR. Topp et al. [3] reported a calibration curve for predicting the volumetric content of water in the soil as a representative application. This method has been extended to various soil textures [4,5,6] to understand soil behavior. In particular, Caron et al. [7] used the gas relative diffusivity, which is the diffusion ratio between given gas and free air in the soil at the same temperature to obtain hydraulic conductivity through TDR and the hydraulic conductivity at certain air entry values was estimated using the Laplace equation under a sorption-desorption process. TDR was used to measure appropriated water content to find the point of air entry in this study. Al-Jabri et al. [8] selected TDR to measure the solution change with different salty concentrations, and the hydraulic conductivity was estimated using the discharge rate and flux density in the wooding equation. The distributions of hydraulic conductivity were addressed across the plant rows and the representative values were selected through the histogram. Liu et al. [9] also used the TDR to obtain volumetric water content in the water retention curve, and the hydraulic conductivity was estimated through Darcy’s law through shape parameters related to water flow. Although a large number of studies were conducted to evaluate hydraulic conductivity through TDR, in previous studies, TDR was mainly used as an auxiliary means to investigate the water content and concentration rate, which are the input parameters of the applied formula. Unlike previous studies, this study is focused on proposing a directly linked method that can calculate the hydraulic conductivity based on the electrical characteristics of the TDR signal through changed energy depending on the soil medium.

The objective of this study is to develop a method for measuring hydraulic conductivity through the dielectric constant. Thus, the theoretical relationship between the dielectric constant and hydraulic conductivity is analyzed. Laboratory tests including the developed cell, electrode, specimen, and measurement procedures are presented. The dielectric constant analysis through measured waveforms and the predicted hydraulic conductivity are explained. The field test is also performed; the measured dielectric constant and the converted hydraulic conductivity are described in detail. Finally, the verification of the proposed method is validated by comparing the hydraulic conductivities deduced by the dielectric constant and constant-head test (reference values).

## 2. Background Theory

### 2.1. Dielectric Constant

**Figure 1:**
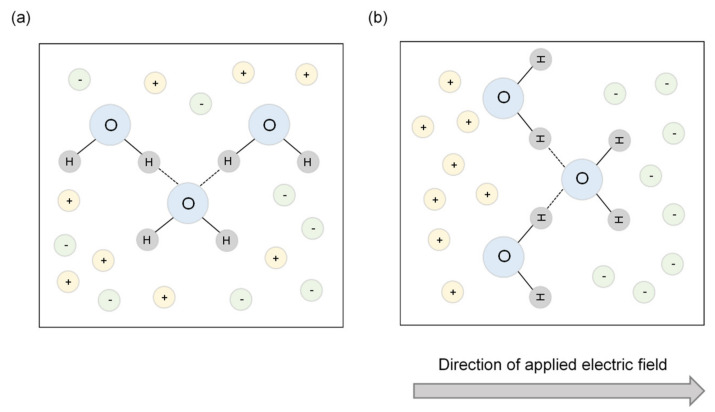
The water molecule with positive and negative charges: (**a**) the natural condition; (**b**) the arranged condition due to the applied electric field. The stored energy of the water molecule is a real part of the dielectric constant, and the energy loss initiated by environmental ions (positive and negative ions) is described as the imaginary part of the dielectric constant. The dotted line shows the hydrogen bond.

The dielectric constant is determined mainly by the Coulomb force in an electric field. The dielectric constant of water is higher than that of soil particles because of the presence of ions; the water molecule has positive and negative charges with a hydrogen bond. It can store energy under the changed electrical conditions when an additional charge is introduced into the material, as shown in Figure 1. Energy loss also occurs owing to the polarization and Ohmic behavior in a porous material [10]; this means that the porous material does not consist only of water molecules. The accumulated and dissipated energies correspond to the real (*ε_r_*) and imaginary (*ε_i_*) parts of the dielectric constant, respectively. The dielectric constant of the porous material (*ε_pm_*) can be mathematically expressed using the electrical conductivity of the material (*σ*), frequency of the applied electric field (f), dielectric constant of free space (*ε_f_*), and dielectric constants *ε_r_* and *ε_i_*. Although Equation (1) shows that the dielectric constant of the material consists of real and imaginary parts, only the real part of the dielectric constant is generally considered for the porous material (soil medium) while neglecting the imaginary part [11].

(1) $$\varepsilon_{pm}=\varepsilon_{r}-i\left(\varepsilon_{i}+\frac{\sigma }{2\pi f\varepsilon_{f}}\right)$$

where i denotes the imaginary number.

When the electric charge is applied in the medium, the electromagnetic wave velocity can be expressed as

(2) $$V=\frac{1}{\sqrt{u_{f}u_{pm}\varepsilon_{f}\varepsilon_{pm}}}$$

where μ and ε denote the magnetic permeability and dielectric constant, respectively. The subscripts “*f*” and “*pm*” correspond to free space and porous material, respectively. The velocity of light (c) is determined by the magnetic permeability and dielectric constant of free space (c^2^ = 1*/μ_f_ε_f_*). Therefore, Equation (2) can be written as

where *μ* and *ε* denote the magnetic permeability and dielectric constant, respectively. The subscripts “*f*” and “*pm*” correspond to free space and porous material, respectively. The velocity of light (*c*) is determined by the magnetic permeability and dielectric constant of free space (*c^2^* = 1/*μ_f_ε_f_*). Therefore, Equation (3) can be written as

(3) $$V=\frac{c}{\sqrt{\varepsilon_{pm}}}$$

where the magnetic permeability of the porous medium is neglected because it is approximately equal to one for most soil materials on earth [12].

TDR is used to transmit an electromagnetic wave and receive the reflected signal. A step voltage is generated through a pulser. The electromagnetic wave propagates through a coaxial cable with conductors and is finally reflected at the tip of the conductor. The velocity can be expressed as *V = 2L/t*, where t is the reflection time and L is the travel length of the conductor. The round-trip distance during the transmission and after the reflection is considered. The velocity deduced by TDR can be substituted in Equation (4); thus, the dielectric constant can be expressed in terms of the properties of the waveform measured by TDR.

(4) $$\varepsilon_{pm}={\left(\frac{ct}{2L}\right)}^{2}$$

### 2.2. Hydraulic Conductivity

The hydraulic conductivity (*K*) is the flow rate of the liquid in the porous medium, which can be expressed by the Kozeny–Carman formula,

(5) $$K=\left(\frac{\gamma }{\mu }\right)\left(\frac{1}{C_{K-C}}\right)\left(\frac{1}{S_{0}^{2}}\right)\left[\frac{e^{3}}{\left(1+e\right)}\right]$$

where *γ*, *μ*, and e denote the unit weight, the viscosity of the liquid, and void ratio, respectively. The ratio γ/μ is approximately equal to 9.93 × 10^4^ cm·s^-1^ at 20 °C [13]. C_K-C_ is the Kozeny–Carman empirical coefficient, which is usually equal to approximately 5.0. S_o_ is the specific surface area per unit volume of particles, which can be calculated by the ratio between the shape factor (SF) and effective diameter (D_eff_). The SFs are in the ranges of 6.0–6.6 and 7.7–8.4 for round and angular particles, respectively. The effective diameter can be deduced through a sieve analysis [14].

### 2.3. Relationship between the Dielectric Constant and Hydraulic Conductivity

**Figure 2:**
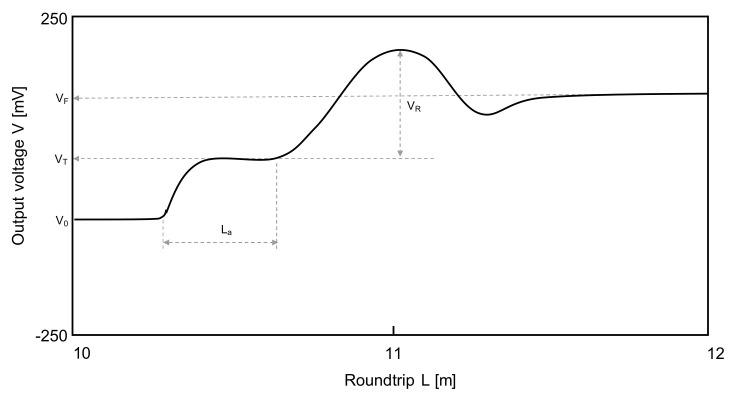
An example of the TDR waveform. V_0_, V_T_, and V_F_ mean initial voltage, first reflected voltage, and converged voltage, respectively. V_R_ denotes the different voltage with the first and second reflection. L_a_ is the distance between the first and second reflected positions.

When an input voltage propagates in the medium, its amplitude is attenuated according to the material conductivity, which enables the measurement of the electrical conductivity of the material through TDR. Dalton et al. [15] reported that the electrical conductivity of a porous medium (*σ_pm_*) is a function of the dielectric constant (*ε_pm_*), length of the TDR electrode (*L*), and voltages of the reflected waveform (V_1_ and V_2_) based on the electromagnetic field theory, as given by Equation (6). Nadler et al. [16] also reported an expression for the electrical conductivity of the porous medium (*σ_pm_*) that improved the limit of Equation (6) with the use of only two voltage values. Even though the resolutions of the deduced electrical conductivity based on Equations (6) and (7) are slightly different, both equations are used in this study owing to their frequent usage and high accuracies [17].

(6) $$\sigma_{pm}=\frac{\sqrt{\varepsilon_{pm}}}{120\pi L}\ln(\frac{V_{T}}{V_{R}})$$

(7) $$\sigma_{pm}=\frac{K_{C}}{Z_{0}(\frac{1+(\frac{V_{F}-V_{0}}{V_{0}})}{1-(\frac{V_{F}-V_{0}}{V_{0}})})-Z_{cable}}f_{t}$$

where K_C_ denotes the cell constant according to the probe, which can be obtained through a calibration test; Z_0_ and Z_cable_ are the impedances of the coaxial and used cables (both Z_0_ and Z_cable_ are 50 Ω in this study because a coaxial cable is used), respectively; V_0_, V_T_, and V_F_ are the voltages at the initial, first, and the final converged points as shown in Figure 2, respectively; V_R_ denotes the voltage difference between the first and second reflection points; f_t_ is generally equal to 1 at 25 °C.

The electrical conductivity (*σ*) of a soil mixture is influenced by the soil particles, electrolyte, and specific surface. When the electrical conductivity of the electrolyte is high, the conductivity of the porous material can be expressed by the electrical conductivity of the electrolyte (*σ_el_*), porosity (*n*), tortuosity factor (*α*), and cementation factor (β) [18],

(8) $$\sigma_{pm}=\alpha \sigma_{el}n^{-\beta }$$

The tortuosity factor and cementation factor generally lie in the ranges of 0.6–3.5 and 1.4–2.2, respectively [19].

The electrical conductivity expressions Equations (6)–(8) can be used to derive relationships containing the porosity (n = e/(1 + e)) determined by the void ratio (e). By substituting the void ratio into Equation (5), the relation between the hydraulic conductivity and dielectric constant can be derived.

(9) $$K\approx F_{KC}\cdot \frac{{\left(F_{EM\_Dalton(Nadler)}\right)}^{3}}{{\left(F_{EM\_Dalton(Nadler)}-1\right)}^{2}}$$

(10) $$F_{KC}=\left(\frac{\gamma }{\mu }\right)\left(\frac{1}{C_{K-C}}\right)\left(\frac{1}{S_{0}^{2}}\right)$$

(11) $$F_{EM\_Dalton}=\exp^{\frac{\ln(L\cdot \frac{V_{T}}{V_{R}})+\ln(\frac{1}{\sigma_{el}}\cdot \alpha )-0.5\ln(\varepsilon_{pm})+5.9}{\beta }}$$

(12) $$F_{EM\_Nadler}=\exp^{\frac{\ln(Z_{cable}+(\frac{\frac{1}{V_{0}}\cdot (V_{F}-V_{0})-1}{\frac{1}{V_{0}}\cdot (V_{F}-V_{0})+1}\cdot Z_{0}+\ln(\frac{1}{\sigma_{el}}\cdot \alpha )-ln(K_{C})}{\beta }}$$

where F_KC_ is deduced by the Kozeny–Carman formula. F_EM_Dalton_ and F_EM_Nadler_ were estimated by Dalton et al. [15] and Nadler et al. [16], respectively. Equation (9) shows that the physical and electrical properties of the porous medium are related.

## 3. Laboratory Test

### 3.1. Specimen

**Figure 3:**
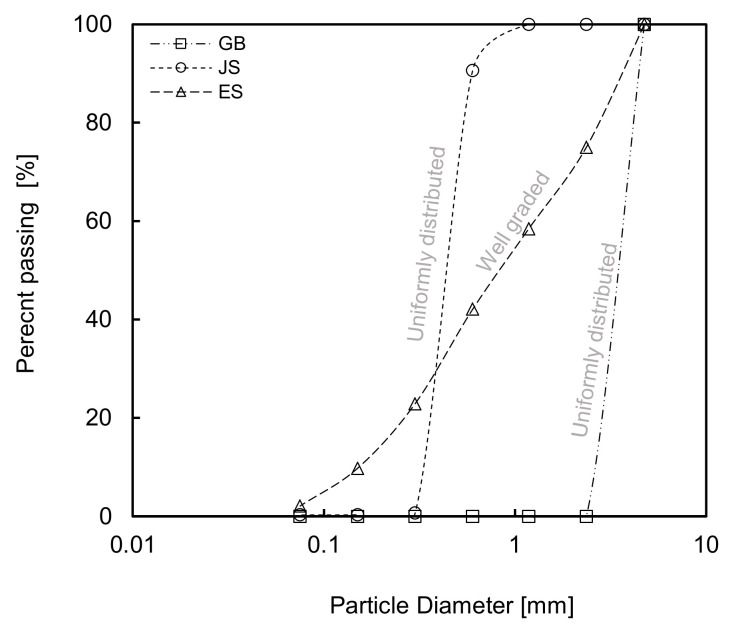
Results of sieve test for glass bead (GB), Jumunjin sand (JS) and extracted soil from field (ES).

Three different granular specimens, namely a glass beads (GB), Jumunjin sand (JS), and soil extracted from field (ES), are used in the laboratory test to verify the proposed method considering different particle characteristics. The particle size distributions are plotted in Figure 3. The median particle sizes (D_50_) of GB, JS, and ES are estimated to be approximately 0.51, 0.76, and 0.95 mm, respectively. Figure 3 shows that GB and JS are uniformly distributed specimens, while ES is a well-graded soil. The physical properties of each specimen are summarized in Table 1.

### 3.2. Cell

**Figure 4:**
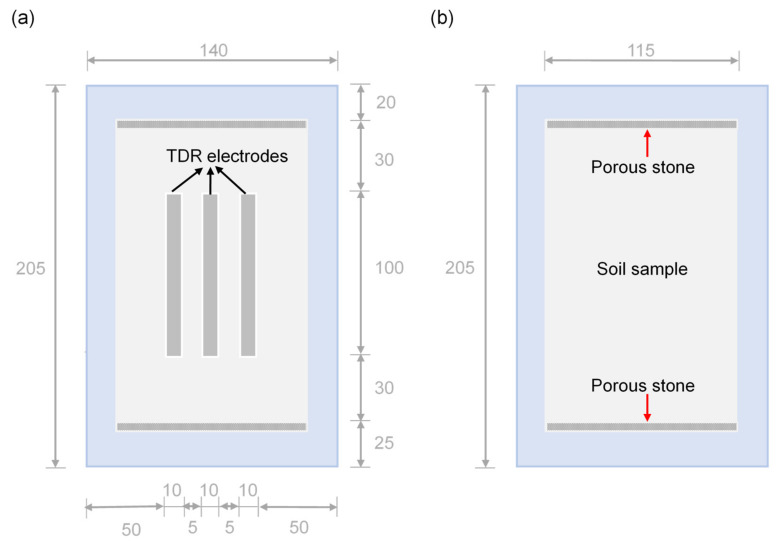
Schematic drawing of the TDR-hydraulic conductivity cell in sectional view: (**a**) the area with TDR electrodes; (**b**) the symmetrically opposite area. The unit of value is mm.

A TDR hydraulic conductivity cell with a cylindrical shape was fabricated to measure both dielectric constant and hydraulic conductivity, as illustrated in Figure 4. The outer diameter of the cell was 140 mm, the inner diameter was 115 mm (for the placement of the sample), and the height was 205 mm. A nonconductive nylon was employed as the cell material to reduce the electrical disturbance in the measurement of the electromagnetic waves. Three electrodes were vertically installed on the wall of the cell to measure the dielectric constant, as shown in Figure 4. They had widths, lengths, and thicknesses of 10, 100, and 2 mm, respectively, and were connected to the coaxial cable. The middle electrode was soldered to the center of the coaxial cable, while the other two electrodes were connected to the external cable. To prevent the disturbance of the specimen due to the protrusion of the electrode into the sample, a groove was formed in the cell with the thickness of the electrode and the electrode was integrated with the cell. In addition, the cell could be used to measure the water head to perform a constant-head experiment. The measured hydraulic conductivity in this experiment was used as the reference value.

### 3.3. Measurement System

The dielectric constant can be estimated through the output voltage, at the medium state. A high-speed signal path analyzer (HYPERLABS, Beaverton, Oregon: HL-1001) was used to apply an input voltage and gather the output signal. The input voltage of 250 mV was transferred to the coaxial cable through the device. Reflection occurred owing to the impedance difference. The non-reflected energy continuously propagated through the electrode to the medium, and finally, the reflected signal was stored. Twenty signals were averaged to reduce the random noise of the electromagnetic wave.

The constant-head test was carried out with circulating fluid (≈1000 mL) using a water tank and pump. The fluid flowed from the bottom to the top. Two manometers were used to measure the water head difference. The hydraulic conductivity was calculated using Darcy’s law. In this experiment, salty water with a concentration of 0.5 M (≈33 mS/cm) was used as the fluid owing to the increased resolution of the electromagnetic wave measurement.

### 3.4. Performance

**Figure 5:**
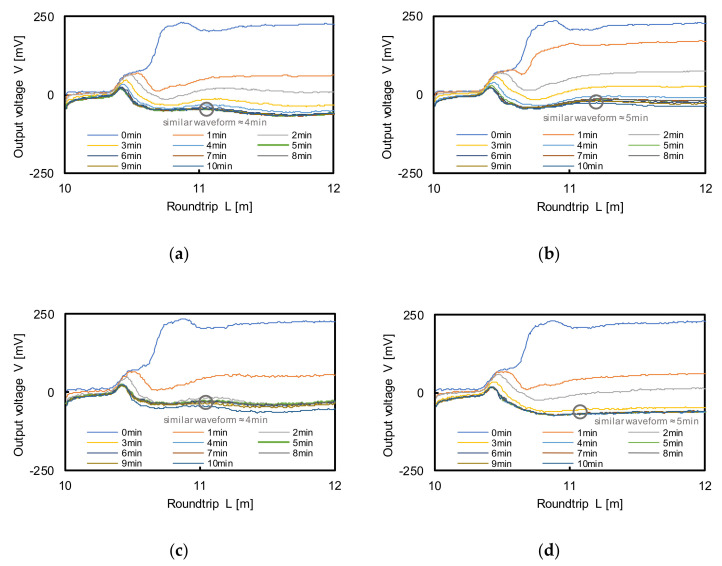

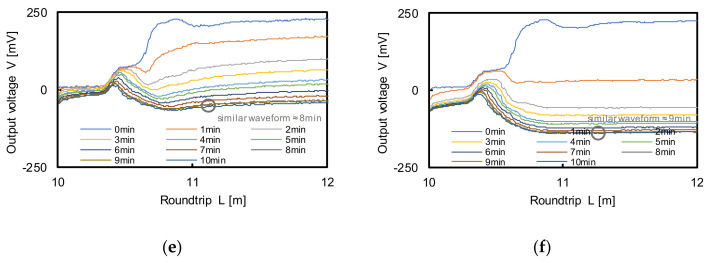
Measured TDR waveforms: (**a**,**b**) glass bead (GB); (**c**,**d**) Jumunjin sand (JS); (**e**,**f**) extracted soil from field (ES). The Dr denotes the relative density. Dr = 40% (**a**,**c**,**e**), Dr = 80 % (**b**,**d**,**f**).

The prepared GB, JS, and ES specimens had two different relative densities, which ratio of the difference between the maximum and natural void ratios to the difference between the maximum and minimum void ratios, of 40% and 80%, used to evaluate the changes in characteristics. The air pluviation method was used to obtain uniform specimens. Preliminary experiments were carried out to determine the circulation time of the fluid to achieve fully saturated specimens. The reflected waveforms in the TDR were measured while circulating the fluid after every 1 min; the results are plotted in Figure 5. With the saturation of the specimens, the first reflection output voltages in the waveforms gradually decreased and then the signals exhibited similar trends after approximately 4, 4, and 8 min for GB, JS, and ES with the relative density of 40% and after approximately 5, 5, and 9 min at the relative density of 80%, respectively. Therefore, the circulation time of 9 min (mean value) was sufficient to achieve a 100%-saturated specimen. However, the circulation time was set to 30 min considering the full-circulation conditions. Then, both the TDR waveform and water head were measured.

## 4. Results

### 4.1. Verification of the Saturation

**Figure 6:**
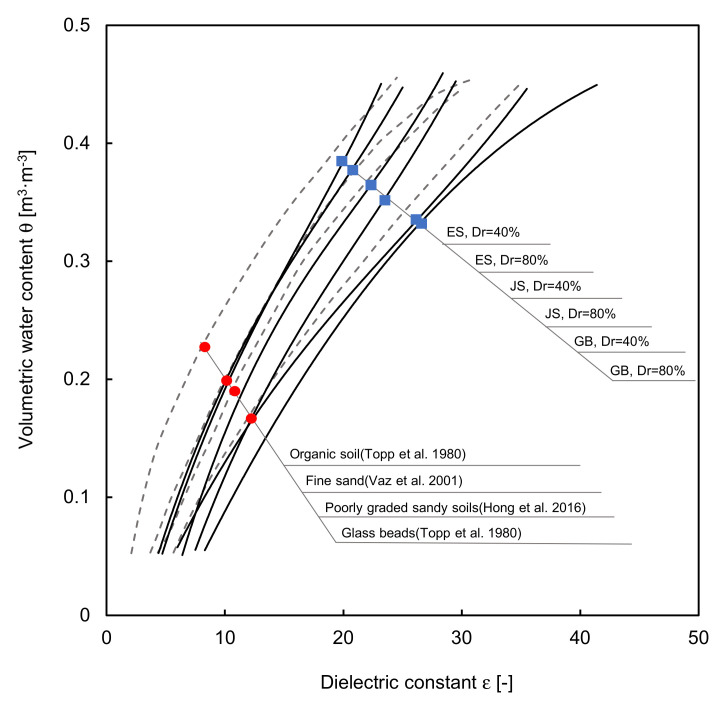
The calibration results between the dielectric constant and saturation. GB, JS, and ES denote the glass beads, Jumunjin sand, and the extracted specimen.

Even though the circulation time was sufficient, it was necessary to evaluate whether the finally measured waveforms corresponded to 100% saturation. A calibration test was performed to obtain the relationships between the saturation and dielectric constant for GB, JS, and ES with the relative densities of 40% and 80%. Figure 6 shows the results, which can be expressed by Equations (13)–(15) and Equations (16)–(18) for GB, JS, and ES with the relative densities of 40% and 80%, respectively.

(13) $$\theta =5\times 10^{-6}\varepsilon^{3}-5\times 10^{-4}\varepsilon^{2}+2.47\times 10^{-2}\varepsilon -7.07\;\times 10^{-2}$$

(14) $$\theta =8\times 10^{-6}\varepsilon^{3}-6\times 10^{-4}\varepsilon^{2}+2.6\times 10^{-2}\varepsilon -7.85\times 10^{-2}$$

(15) $$\theta =1\times 10^{-6}\varepsilon^{3}-3\times 10^{-4}\varepsilon^{2}+2.47\times 10^{-2}\varepsilon -12.98\times 10^{-2}$$

(16) $$\theta =0.3\times 10^{-6}\varepsilon^{3}-19\times 10^{-4}\varepsilon^{2}+5.38\times 10^{-2}\varepsilon -22.33\times 10^{-2}$$

(17) $$\theta =0.2\times 10^{-6}\varepsilon^{3}-13\times 10^{-4}\varepsilon^{2}+4.32\times 10^{-2}\varepsilon -20.39\times 10^{-2}$$

(18) $$\theta =20\times 10^{-5}\varepsilon^{3}-62\times 10^{-4}\varepsilon^{2}+9.42\times 10^{-2}\varepsilon -17.94\times 10^{-2}$$

where θ is volumetric water content [m^3^⋅m^−3^] which is the ratio of water volume to soil volume. The ε denotes dielectric constant [-].

The results are similar to those in previous studies (dotted line in Figure 6), which follow the cubic trend and are in a similar range [3,20,21]. If the relative density is low, the increase in saturation is relatively fast, and thus the slope of the calibration curve is steep. In the case of ES, a well-graded soil, various grain particles are mixed, and the difference in the slope of the calibration curve between the samples with the two different relative densities is larger.

### 4.2. Electrical Conductivity

**Figure 7:**
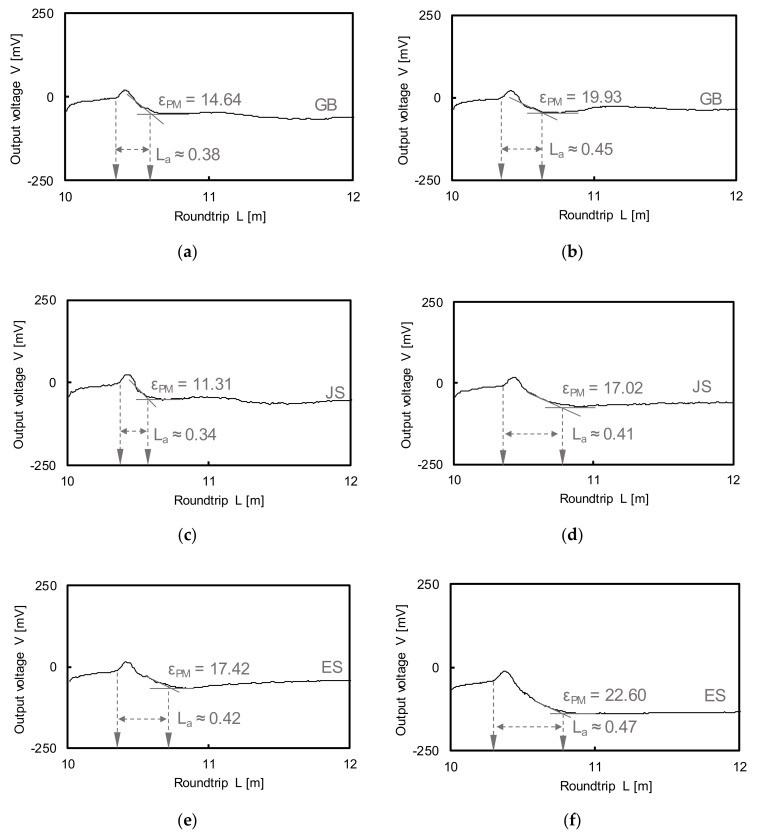
Waveforms of each specimen with dielectric constants: (**a**,**c**,**e**) relative density of 40%; (**b**,**d**,**f**) relative density of 80%.

The measured waveforms at full saturation are presented in Figure 7. The dielectric constant is calculated using the diagram presented by Topp et al. [3]. At the relative density of 40%, the calculated dielectric constants of GB, JS, and ES were 14.64, 11.31, and 17.42, while those at the relative density of 80% were 19.93, 17.02, and 22.60, respectively. With the increase in the degree of compaction, the amount of air inside the voids decreased. Considering that the dielectric constant of air is approximately 1 at 25 °C, the achieved large dielectric constant is attributed to the low amount of air at 80%.

**Figure 8:**
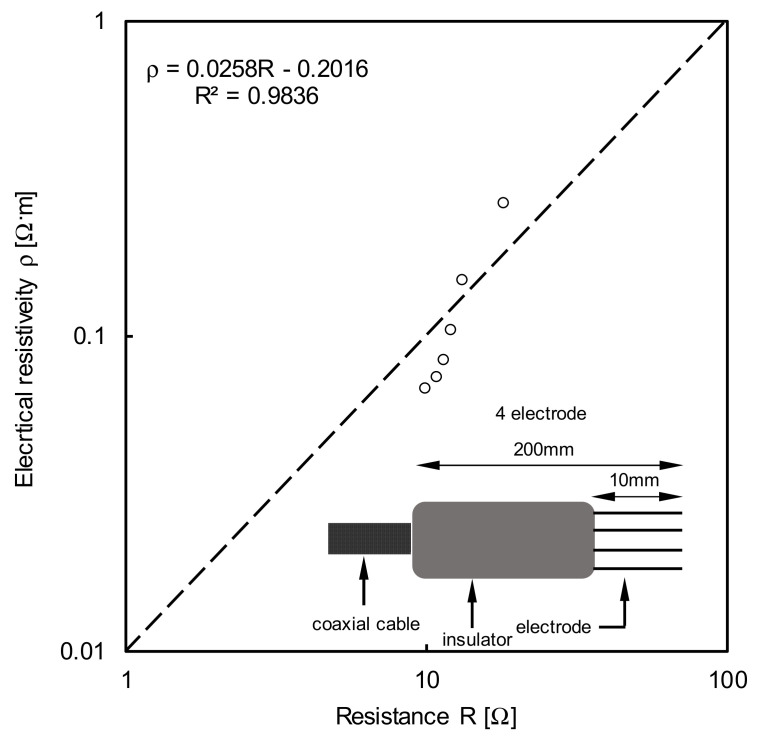
The calibration result of the 4 electrode resistivity probe (4ERP).

The electrical conductivity was deduced based on Equation (5), proposed by Dalton et al. [15]. The method of Nadler et al. [16] requires the cell constant, and thus a 4 electrical resistivity probe (4ERP) experiment is performed to obtain the true electrical conductivity. A schematic of the 4ERP setup including the electrodes, insulator, and coaxial cable is presented in Figure 8. The experiment was performed at six different concentrations of the solution. The 4ERP method was reported in detail by Kim et al. [22]. Figure 8 shows the 4ERP calibration results. The relationship between the electrical resistivity and resistance is almost linear with a coefficient of determination of 0.9836.

**Figure 9:**
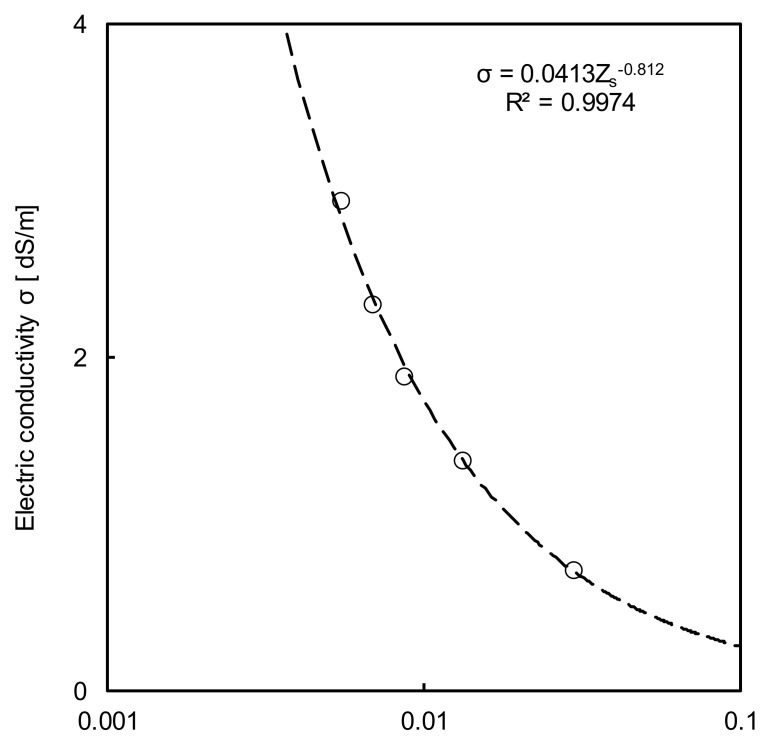
The relationship between electrical conductivity and impedance of the soil sample for obtaining cell constant. The cell constant is deduced as 0.0413 in this study.

**Figure 10:**
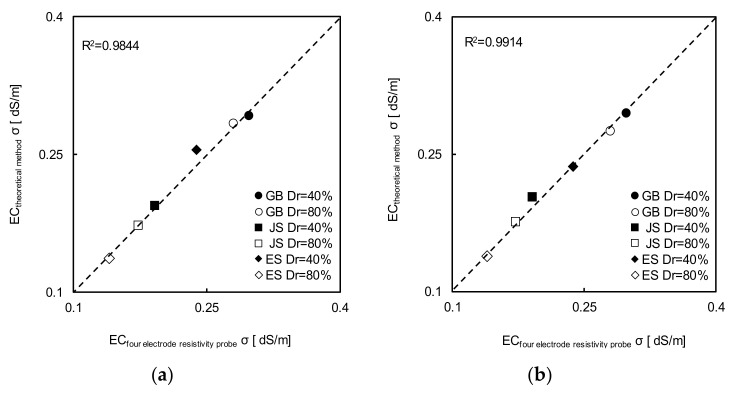
A comparison of the electrical conductivity deduced by 4ERP and the theoretical method based on: (**a**) Equation (11); (**b**) Equation (12). Note that EC denotes the electrical conductivity.

The relationship between the electrical conductivity and impedance of the sample is presented in Figure 9. The electrical conductivity was measured using the 4ERP method as a true value. The TDR hydraulic conductivity cell was used to measure the impedances of the soil samples with four different concentrations. The obtained cell constant was 0.0413, as shown in Figure 9. The electrical conductivity, obtained by the method of Nadler et al. [16], is presented in Figure 10. The electrical conductivities calculated using the methods of Dalton et al. [15] and Nadler et al. [16] were similar to that measured using the 4ERP method with a high coefficient of determination. These results show that the electrical conductivity calculation is highly reliable.

### 4.3. Hydraulic conductivity

**Figure 11:**
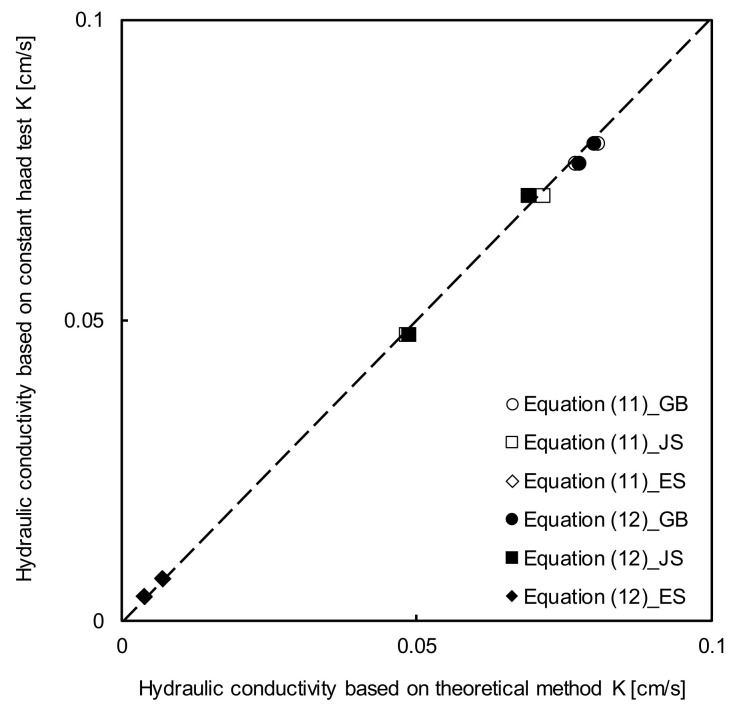
Comparison of hydraulic conductivity obtained by the theoretical methods and constant head test.

The tortuosity factor (α) was assumed to be unity [19], while the cementation factor (β) of GB, JS, and ES were 1.3, 1.1, and 2.3, respectively, considering the particle interactions [22,23] in Equations (11) and (12). The parameters were determined using the measured results, and then the hydraulic conductivity was calculated as shown in Equation (9); the results are shown in Figure 11. The hydraulic conductivity determined by the constant-head test is also presented as the true value in Figure 11. The hydraulic conductivities derived by the TDR and constant-head test were in the ranges of approximately 0.00402–0.08065 cm/s and 0.00407–0.07946 cm/s, respectively. Assuming the value obtained by the constant-head test as the true value, the calculated error ratios of GB, JS, and ES were 1.5%, 1.1%, and 3.5% at the relative density of 40%, while those at the relative density of 80% were similar, 1.1%, 1.1%, and 1.2%, respectively. The small error ratio shows that the hydraulic conductivity can be estimated using the dielectric constant with high reliability.

## 5. Discussion

### 5.1. Sensitivity

**Figure 12:**
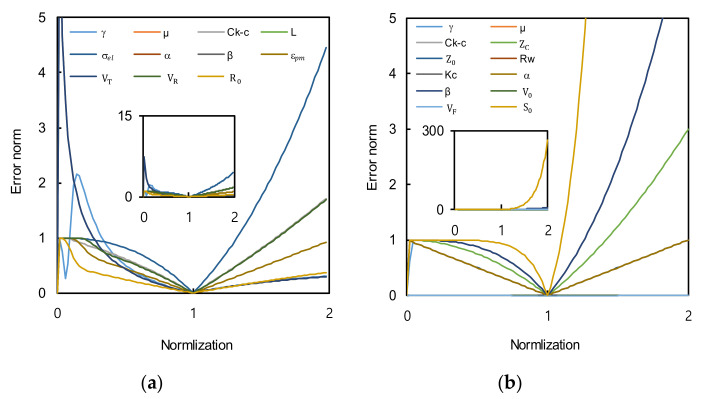
The sensitivity of every input parameter in theoretical methods based on: (**a**) Dalton et al. [15]; (**b**) Nadler et al. [16].

The theoretical methods for the calculation of the hydraulic conductivity based on Equations (11) and (12) involve 11 and 12 variables, respectively. It is difficult to correctly gather all input parameters, and thus a sensitivity analysis of each parameter was performed. The sensitivity was calculated using the hydraulic conductivity changes when the selected input parameter was increased and decreased within a reasonable range. If the hydraulic conductivity change is large, the influence of the selected input value is large. The input value is normalized based on the reference value of each parameter for comparison because the input values are in various ranges. When the normalized value is 1, the sensitivity is 0 because there is no change. The reference value of each input parameter is determined by considering the measured values in Figure 11 and presented in Table 2. The sensitivities of the calculations by Equations (11) and (12) are presented in Figure 12. For Equation (11), when the input value is larger than the reference value, the parameters can be ordered by their sensitivities as σ_el_ > β ≈ V_R_ ≈ C_K-C_ ≈ μ > ε_pm_ > S_0_ > V_T_ ≈ L ≈ γ ≈ α. In the opposite case, when the input value is below the reference value, the sequence is V_T_ ≈ α ≈ L > γ > σ_el_ > C_K-C_ ≈ _μ_ ≈ V_R_ ≈ β > ε_pm_ > S_0_. For Equation (12), the parameters affecting the hydraulic conductivity can be ordered as S_0_ > β ≈ K_C_ ≈ C_K-C_ ≈ μ > Z_C_ > _α_ ≈ R_W_ ≈ Z_0_ ≈ γ > V_0_ ≈ V_F_ regardless of the ranges of the normalized values. The influencing factors are summarized in Table 3. Most parameters should be carefully measured because the input factors of each equation are determined mainly by measurements. In particular, σ_el_, V_T_, and S_0_ exhibited the largest influences, which shows that the characterization of the medium and TDR waveform are the most important for the calculations. α and β, which should be determined using literature values, are significant parameters, and thus a careful analysis is required prior to the use of assumptions. α is mainly assumed to be unity in the unconsolidated state [19]. However, β has various values of 1.3, 1.6, 2.15, and 5.12 for glass sphere, natural sand, rock, and sandstone, respectively [24,25,26,27,28], as is affected by the pore structure, compaction, and grain type. The value of β should be chosen considering the ground conditions. Additional calibration is required to select a reasonable value.

### 5.2. Field Application

**Figure 13:**
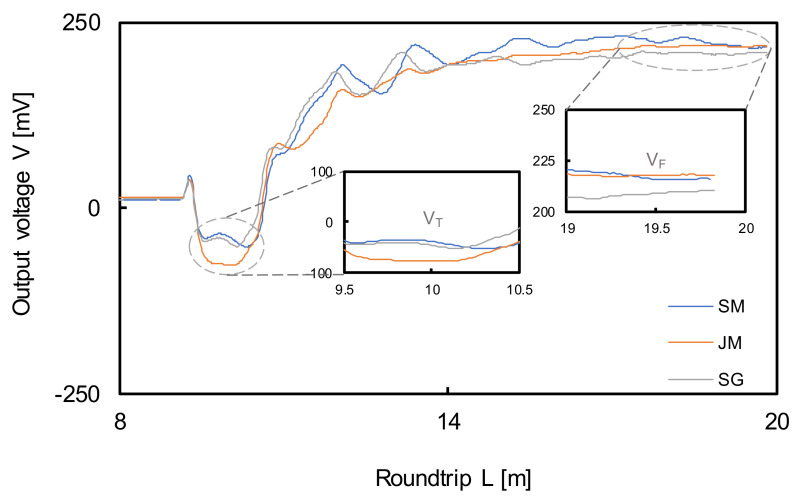
The measured TDR waveforms in-field.

**Figure 14:**
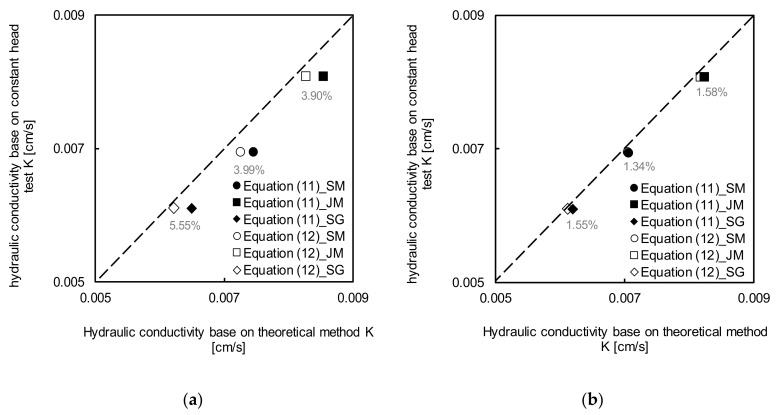
Comparisons of hydraulic conductivity obtained by constant head test and theoretical methods with cementation factor of: (**a**) 1.5; (**b**) 2.0.

To evaluate the proposed method, field experiments were carried out in three river beds, namely Sikjang mountain (SM), Jangnyeong mountain (JM), and Sutonggol (SG), in Daejeon, South Korea, as fully saturated regions. The reflected waveforms were recorded by the TDR system as shown in Figure 13. The initial voltages (V_0_) were the same (approximately 10 mV) in all regions. However, the first reflection voltages for the SM, JM, and SG were −50, −60, and −50 mV, while the final reflection voltages were approximately 220, 222, and 210 mV, respectively. To obtain the input parameters in (11) and (12), disturbed specimens were extracted from the local area where the field tests were performed. The characterization results for each specimen are summarized in Table 4. A constant-head test was also performed on the extracted specimens. The obtained hydraulic conductivities for SM, JM, and SG were 0.006946 cm/s, 0.008077 cm/s, and 0.006103 cm/s, those estimated by (11) were 0.007256 cm/s, 0.008537 cm/s, and 0.006503 cm/s, and those estimated by (12) were 0.007256 cm/s, 0.008537 cm/s, and 0.006503 cm/s, respectively. The same input parameters were used as in the laboratory tests. The hydraulic conductivities are compared in Figure 14. The average error ratios for SM, JM, and SG calculated using (19) were 3.99%, 3.90%, and 5.55%, respectively. The low error ratio shows the high reliability of the method.

(19) $$\mathrm{Error}\;\mathrm{ratio}=\frac{k_{\mathrm{inference}}-k_{\mathrm{true}}}{k_{\mathrm{true}}}$$

The cementation factor, the parameter with the highest sensitivity in (11) and (12), was adjusted to reduce the error ratio. The average median particle size (D_50_) of the field samples was ≈850% larger than that of the specimen analyzed in the laboratory test. Therefore, the grain and pore sizes of the field specimen were larger, and thus the cementation factor was also larger. A higher cementation factor of 2.0 was chosen considering the reported values [29]. Based on the determined cementation factor, the hydraulic conductivity was estimated. The average error ratio was reduced to approximately 1.49%, as shown in Figure 14b.

These results show that the hydraulic conductivity can be immediately calculated in the field by the proposed method. A more reliable hydraulic conductivity can be derived if the cementation factor, among the input variables, is determined considering the sample conditions.

## 6. Conclusions

The aim of this study was to propose an improved method for measuring hydraulic conductivity that overcomes the shortcomings of the existing method. The conclusions of this study can be summarized as follows:

- The electrical resistivity of the medium is related to hydraulic conductivity. The electrical conductivity, which is the reciprocal of electrical resistivity, can be estimated by the dielectric constant through the attenuating amplitude of the output voltage.
- Two theories were used to deduce the relationship between the dielectric constant and hydraulic conductivity. The proposed methods were verified by the laboratory test. A reasonable hydraulic conductivity was estimated based on a comparison with a reference value obtained by the constant-head test.
- The proposed equations had many input parameters; the influence of each parameter was investigated through the error-norm technique. Among the various variables, the cementation factor exhibited a high sensitivity; thus, a careful analysis is required for determining the cementation factor.
- The proposed methods were verified through the field test. They can be reliably used to estimate the hydraulic conductivity with high resolution even under field conditions.
- This study is focused on samples with large particle size, and thus, it has an advantage of high reliability when the fine contents are small. However, it is considered that the attenuation of the TDR signal will be different when the soil characteristics are changed in the local area due to the content of fine particles. Further research is needed to reasonably improve the characteristics of the TDR probe, including the diameter of the electrode, penetrated length, and input voltage to increase resolution of the proposed equation in various soil mediums.

## Figures and Tables

**Table 1:** Properties of glass beads, Jumunjin sand, and extracted soil from field.

	Glass Beads (GB)	Jumunjin Sand (JS)	Extracted Soil from Field (ES)
Maximum void ratio (e_max_)	0.68	0.89	0.82
Minimum void ratio (e_min_)	0.63	0.81	0.59
Specific gravity	2.62	2.61	2.67
Median particle size (D_50_)	0.51 (mm)	0.76 (mm)	0.95 (mm)
Coefficient of uniformity (C_u_)	1.50	1.49	6.50
Coefficient of curvature (C_c_)	1.09	1.01	1.16

**Table 2:** The reference values for performing the sensitivity analysis.

Theoretically Derived Equation (11)		Theoretically Derived Equation (12)	
γ (kN/m^3^)	9.798	γ (kN/m^3^)	9.798
μ (mPa⋅s)	1.002	μ (mPa⋅s)	1.002
C_K-C_	5	C_K-C_	5
S_0_ (mm)	2000	S_0_ (mm)	2000
L (m)	10	Z_C_ (Ω)	50
σ (S/m)	0.002923	Z_0_ (Ω)	50
α	1	R_W_ (Ω⋅m)	0.003356
β	1.3	K_C_	0.04313
ε	17.4295	α	1
V_T_ (mV)	210	β	1.3
V_R_ (mV)	15	V_0_ (mV)	248
-	-	V_F_ (mV)	204

**Table 3:** The reference values for performing the sensitivity analysis.

Theoretically Derived Equation (11)					Theoretically Derived Equation (12)			
	Input Value > Reference Value		Input Value < Reference Value		Input Value > Reference Value		Input Value < Reference Value	
High 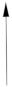 Sensitivity 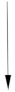 Low	σ_el_ (S/m)	M	V_T_ (mV)	M	S_0_ (mm)	M	S_0_ (mm)	M
	V_R_ (mV)	M	α	C	β	A	β	A
	β	A	L (m)	M	K_C_	M	K_C_	M
	C_K-C_	A	γ (kN/m3)	M	C_K-C_	A	C_K-C_	A
	μ (mPa⋅s)	M	σ_el_(S/m)	M	μ (mPa⋅s)	M	μ (mPa⋅s)	M
	ε_pm_	M	C_K-C_	A	Z_C_ (Ω)	C	Z_C_ (Ω)	C
	S_0_ (mm)	M	μ (mPa⋅s)	M	α	C	α	C
	V_T_ (mV)	M	V_R_ (mV)	M	R_W_ (Ω⋅m)	M	R_W_ (Ω⋅m)	M
	L (m)	M	Β	A	Z_0_ (Ω)	M	Z_0_ (Ω)	M
	γ (kN/m3)	M	ε_pm_	M	γ (kN/m^3^)	M	γ (kN/m^3^)	M
	α	C	S_0_ (mm)	M	V_0_ (mV)	M	V_0_ (mV)	M
					V_F_ (mV)	M	V_F_ (mV)	M

M: Measured value, C: Constant, A: Assumed value.

**Table 4:** The properties of field soils extracted from Sikjang mountain (SM), Jangnyeong mountain, (JM) and Sutonggol (SG).

	Sikjang Mountain (SM)	Jangnyeong Mountain (JM)	Sutonggol (SG)
Maximum void ratio (e_max_)	0.93	0.89	0.82
Minimum void ratio (e_min_)	0.75	0.71	0.73
Specific gravity	2.63	2.65	2.65
Median particle size (D_50_)	8.20 (mm)	4.20 (mm)	8.50 (mm)
Coefficient of uniformity (C_u_)	6.70	9.10	6.50
Coefficient of curvature (C_c_)	1.40	2.20	1.25
